# Tunable/Reconfigurable Metasurfaces: Physics and Applications

**DOI:** 10.34133/2019/1849272

**Published:** 2019-07-07

**Authors:** Qiong He, Shulin Sun, Lei Zhou

**Affiliations:** ^1^State Key Laboratory of Surface Physics and Key Laboratory of Micro and Nano Photonic Structures (Ministry of Education) and Department of Physics, Fudan University, Shanghai 200438, China; ^2^Shanghai Engineering Research Center of Ultra-Precision Optical Manufacturing, Green Photonics and Department of Optical Science and Engineering, Fudan University, Shanghai 200433, China

## Abstract

Metasurfaces, ultrathin metamaterials constructed by planar meta-atoms with tailored electromagnetic (EM) responses, have attracted tremendous attention due to their exotic abilities to freely control EM waves. With active elements incorporated into metasurface designs, one can realize tunable and/or reconfigurable metadevices with functionalities controlled by external stimuli, opening a new platform to dynamically manipulate EM waves. In this article, we briefly review recent progress on tunable/reconfigurable metasurfaces, focusing on their working mechanisms and practical applications. We first describe available approaches, categorized into different classes based on external stimuli applied, to realize* homogeneous* tunable/reconfigurable metasurfaces, which can offer uniform manipulations on EM waves. We next summarize recent achievements on* inhomogeneous* tunable/reconfigurable metasurfaces with constitutional meta-atoms locally tuned by external knobs, which can dynamically control the wave-fronts of EM waves. We conclude this review by presenting our own perspectives on possible future directions and existing challenges in this fast developing field.

## 1. Introduction

Metamaterials (MTMs), artificial materials composed by subwavelength metallic/dielectric micro/nanostructures (e.g., “meta-atoms”) with predesigned electromagnetic (EM) responses, have attracted intense research interest in the past decades due to their exceptional abilities to manipulate EM waves. These strongly enhanced wave-control capabilities, typically induced by EM resonances possessed by constitutional meta-atoms, lead to many fascinating physical effects and versatile applications not achievable with naturally occurring materials, such as negative refraction, superimaging, and invisibility cloaking [1–3]. However, many MTMs realized so far exhibit complicated structures, bulky sizes, and relatively high losses (especially in the optical regime), which are unfavorable for practical applications.

To overcome the above issues, metasurfaces, ultrathin MTMs constructed by planar meta-atoms with tailored EM responses arranged in certain sequences, were recently proposed. In sharp contrast to conventional MTMs whose functionalities critically reply on phase accumulations of EM waves propagating inside bulk media, metasurfaces fully exploit the abrupt and controllable phase changes at the structure surfaces by engineering the interactions between EM waves and meta-atoms. Arranging meta-atoms with desired properties in certain predesigned sequences, one can realize metasurfaces exhibiting arbitrary phase/amplitude profiles for reflected/transmitted EM waves, which can efficiently reshape EM wave-fronts based on Huygens' principle, generating new physical effects such as anomalous light reflection/refraction, surface wave couplings, flat-lens imaging, and others [4–12]. In addition to exploring new physics based on these planar structures, scientists also made remarkable achievements on implementing metasurfaces in practical applications, thanks to their unique properties such as low-loss, ease of fabrication/integration, and flat configuration.

Along with the fast development of metasurface research, people gradually realized the limitations of passive metasurfaces in realistic applications, i.e., limited working bandwidths and fixed wave-manipulation functionalities. Therefore, many efforts have been devoted to making tunable/reconfigurable metasurfaces that can actively control EM waves upon external tunings [13–23]. Such attempts are far from easy, however, due to the challenges in finding appropriate strategies to make tunable/reconfigurable meta-atoms at given frequency domains and significantly increased difficulties in locally tuning individual meta-atoms inside a metadevice. Nevertheless, these challenges, together with the pressing demands from application side, drive this field move forward quickly and have made it a frontier in metasurface research.

In this article, we briefly review the key achievements in this fast-developing subfield, focusing particularly on established tuning mechanisms and practical applications of active metasurfaces. We hope such a concise review can serve as a useful guide to help newcomers jump into the field quickly. This review is organized as follows. In Section 2, we introduce different tuning approaches, categorized into different classes based on the external stimuli adopted with some representative implementations, to realize tunable/reconfigurable homogeneous metasurfaces at different frequency regimes, which can actively control EM waves in a uniform manner. We next describe in Section 3 recent efforts of making tunable/reconfigurable inhomogeneous metasurfaces/metadevices with constitutional meta-atoms individually tuned by external knobs, which can realize more sophisticated dynamical manipulations on EM waves. Finally, we conclude this review with our own perspectives on future developments and challenges in this subfield.

## 2. Homogeneous Tunable/Reconfigurable Metasurfaces

We start from introducing the available mechanisms to realize tunable/reconfigurable metasurfaces in which all meta-atoms are tuned by external knobs in a uniform manner. Obviously, the most crucial design step is to combine meta-atoms with appropriate active elements whose EM responses sensitively depend on appropriate external stimuli. In contrast to bulk MTMs involving complex 3D structures, the planar and ultrathin configurations of metasurfaces greatly facilitate the integrations of meta-atoms with different types of active elements. In this section, we categorize recently realized active metasurfaces into different classes based on external stimuli applied (e.g., electrical, optical, mechanical, or thermal stimuli) to control their EM responses. We emphasize that the mechanisms presented below actually provide a solid basis for constructing* inhomogeneous* active metasurfaces with all meta-atoms individually controlled, which can be used to realize more performant active metadevices (see Section 3). 

### 2.1. Electrically Tunable Metasurfaces

Over the past decades, electrically tunable metasurfaces (ETMs) have attracted enormous research interests. Thanks to the maturities of electronic and semiconductor technologies, integrating meta-atoms with different electrically sensitive materials (such as varactor/PIN diodes, liquid crystals (LCs), doped semiconductors, 2-dimenisonal (2D) materials, and conducting transparent metals) has become possible, allowing the realizations of many high-performance active metadevices with functionalities tuned dynamically in different frequency domains.

In the microwave regime, ETMs can be constructed with meta-atoms incorporated with varactor/PIN diodes, whose EM characteristics (say, capacitances) can be dramatically tuned through varying applied voltages. For example, Xu* et al.* experimentally demonstrated a microwave polarization-manipulator in metal/insulator/metal (MIM) configuration, based on meta-atoms with PIN diodes incorporated. By tuning the working state of the incorporated diode from “on” state to “off” one through changing the bias voltage, the authors can dramatically tune the EM responses of the meta-atoms and, in turn, switch the functionality of the device from a EM-wave helicity convertor/hybridizer within two separate frequency bands to a helicity keeper within an ultrawide frequency band [54]. Based on similar concept, versatile dynamic microwave metadevices were realized with different functionalities, such as switchable absorbers [55, 56] and tunable polarization controllers [57].

**Figure 1:**
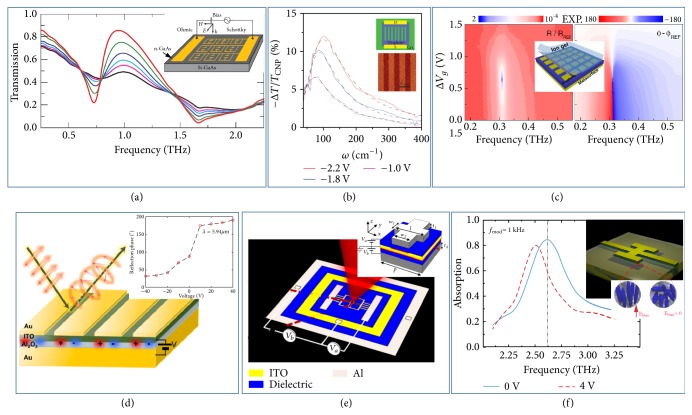
*Electrically tunable metasurfaces.* (a) Switching performance of semiconductor-hybridized ETMs in THz regime. Inset: schematics of the designed metadevices. (b) Plasmon-resonance tuning on graphene microribbon array achieved by varying the electrical gating voltage applied across the graphene ribbons. Insets: metadevice configuration and image of the fabricated graphene ribbons. (c) Wide-range phase modulations on THz waves achieved with electrically gated MIM graphene metasurfaces. Inset: schematics of the MIM graphene metasurface. (d) Dynamic reflection-phase control on IR light achieved with an MIM ETM incorporated with ENZ materials (ITO). Inset: phase of reflected IR light at 5.94 um under different bias voltages. (e) An ITO-involved dual-gated ETM for wide-range reflection-phase modulation (>300°) at 1550 nm. Inset: schematics of the meta-atom design. (f) THz tunable meta-absorber based on MIM meta-atoms incorporated with liquid crystals. Inset: structure of the building meta-atom and the operational principle. Reproduced with permissions: (a) from [24] ©2006 NPG; (b) from [25] ©2011 NPG; (c) from [26]; (d) from [27] ©2017 ACS; (e) from [28] ©2018 ACS; (f) from [29] ©2013 APS.

However, such a design strategy cannot work at frequencies higher than GHz due to lack of suitable varactor diodes. Fortunately, doped semiconductors can be alternative active elements for designing tunable metadevices in the terahertz (THz) and infrared (IR) regimes, since their conductivities (strongly related to their optical responses) can be efficiently modified via charge-carrier doping [24, 58]. In addition, such approach features high modulation speed, broad bandwidth, large dynamic range, and compatibility to complementary-metal-oxide-semiconductor (COMS) technology, thus exhibiting nice potential for real applications. The first experimental demonstration of THz ETM was achieved by Chen* et al.* in 2006 [24]. The realized metadevice was composed by an array of electrically connected gold double split-ring resonators (SRRs), patterned on a thin n-type gallium arsenide (GaAs) layer grown on a semi-insulating GaAs wafer. Varying the external voltage applied across the Schotty diode formed at the metal-semiconductor interface, the authors can dramatically change the carrier density in the n-doped GaAs layer, which, in turn, helps control the THz wave transmitted through the device. As shown in Figure 1(a), a transmission modulation of 50% was experimentally demonstrated at 0.72 THz, dictated by the voltage-controlled conductivity of the doped GaAs layer [24]. By simply optimizing the design of top SRRs, Chen* et al*. further improved the performance of such a THz metasurface, achieving real-time active amplitude (55%) and phase (0.56 rad) modulations with a speed up to 2 MHz for the transmitted beam at 0.81 THz [58]. Further improvements in terms of modulation depths, speeds, or tuning ranges were reported in many literatures where versatile tunable metadevices are realized based on similar mechanism but with different geometries (e.g., metagrating [52]; nanodisk [59]) and active materials (e.g., 2D electron gas systems, epsilon-near-zero (ENZ) materials [52, 59–61], and quantum-well [62]).

Graphene, a 2D material with only one atomic layer, is another excellent candidate to help realize ETMs in THz and mid-IR regimes [63, 64], thanks to its wide-range electrically tunable conductivity. The gate-tunability of graphene is much larger than traditional bulk materials, owing to the low carrier concentration and unique linear band structure of graphene [65]. In 2011, Ju* et al.* experimentally demonstrated that the plasmon resonance of a graphene microribbon array can be dramatically tuned via varying the gating voltage applied [25], as shown in Figure 1(b). Following this seminal work, many other graphene metastructures, ranging from nanorods to disk and inverse-hole arrays, have been subsequently reported, all exhibiting tunable optical responses dictated by the gate-controlled plasmonic resonances in such systems [66–68]. However, the absolute optical responses of these patterned graphene structures are too weak for real applications, since graphene has only one atomic layer and thus its interaction with light is much weaker than bulk materials. To overcome this shortcoming, scientists proposed to combine gate-controlled graphene with appropriately designed metasurfaces to form graphene metasurfaces, which possess both high electrical tunability and strong optical responses. The first experimental demonstration of graphene metasurface was reported by Lee* et al.* in 2012, who successfully integrated a monolayer graphene with an electric metasurface formed by an array of hexagonal metal patches [69]. The electric resonances in the metallic structure strongly enhance the local fields experienced by graphene, which, under external gating, can tune the whole structure's EM responses efficiently, yielding an active amplitude (47%) and phase (32.2°) modulation on EM-wave transmission at 0.86 THz. Inspired by this work, many other graphene metasurfaces were proposed to achieve diversified functionalities in both THz [70–74] and IR regimes [75–77], such as amplitude modulators [71, 72, 75], polarization controller, and phase modulators [73, 76].

However, the phase tuning ranges of these graphene metasurfaces are unfortunately quite narrow (typically much less than *π*), which seriously restrict their applications for dynamical wave-front manipulations. This is not surprising, since the single-layer electric MTMs previously adopted to construct the graphene hybridized structures typically exhibit narrow phase variation ranges, dictated by their Lorentz-type responses. Recently, Miao* et al*. proposed a new mechanism to achieve wide-range active phase control on EM waves based on a new type of graphene metasurfaces [26]. Different from previously realized structures, here the authors chose to combine graphene with an MIM metasurface supporting a magnetic resonance, which exhibits a 2*π* phase variation across its resonant frequency [26]. The underlying physics is based on a generic phase diagram for MIM reflective metasurfaces established with coupled-mode theory [78], which shows that the MIM system behaves as an underdamped (overdamped) oscillator as its radiation quality (Q) factor is smaller (larger) than its absorption Q factor. Interestingly, two different types of oscillators exhibit distinct phase variation behaviors as frequency changes. Graphene, under external gating, essentially functions as a tunable loss to drive the whole metasystem from an underdamped resonator to an overdamped one, thus inducing dramatic changes on the reflection phase accompanying such transition. The authors experimentally demonstrated an absolute phase modulation of ±*π* around the resonant frequency of 0.31 THz by tuning the applied voltage on graphene, as shown in Figure 1(c). Combining two gate-controlled graphene meta-atoms with slightly different resonant frequencies, an extremely large phase modulation (with a maximum phase tuning range of 243°) was experimentally achieved within the frequency interval between the two resonances (at 0.48 THz). Utilizing a similar mechanism, Sherrott* et al.* experimentally demonstrated a maximum phase modulation of 237° at an operating wavelength of 8.5 *μm*, based on reflective graphene metasurface [79] similar to that proposed in [67]. However, the wide-range phase tunings in such metadevices are strongly coupled with amplitude modulations of reflection. In particular, at frequencies close to the resonances, while such metadevices can indeed exhibit large phase modulations (approaching ±*π*), their reflectance also unfortunately turns close to zero, since the underdamped to overdamped resonator transition unavoidably passes through a critical point where perfect absorption occurs. As a result, such strongly diminished reflectance places an obstacle to realize efficient wave-control metadevices except tunable perfect absorbers [71, 77, 80]. In addition to graphene, many other 2D materials (e.g., MoS2, Black Phosphorus, and h-BN) have also been widely used to achieve ETMs at different frequencies for diversified applications [81].

ENZ thin films, made by doped semiconductors or transparent conducting oxide (TCO) materials, have also been combined with passive metasurfaces to realize ESTs in IR and optical regimes. As the permittivity of a material approaches zero, the density of optical modes inside it is strongly enhanced. As a result, optically tuning an ENZ material is much more efficient than tuning other materials. In 2013, Jun* et al*. experimentally demonstrated a gate-tunable strong-coupling effect in a system containing an array of metallic SRRs patterned on a semiconductor substrate possessing an ENZ layer (30 nm n-doped GaAs). Dynamically modulated strong-coupling effect was observed as the ENZ layer was gated electrically, illustrated by a clear resonance frequency shift in the transmission spectrum [60]. Recently, Atwater's group and Brongersma's group realized gate-tunable MIM metasurfaces in near-IR and mid-IR regimes, respectively. In both systems, dielectric spacers in the fabricated MIM metasurfaces were replaced by ENZ layers, which, under external gating, can help tune the EM properties of the whole devices dramatically. By changing the doping conditions of the ENZ layers, the authors could drive their metasystems from underdamped resonators to overdamped ones based on the mechanism described in Ref. [78] and experimentally demonstrated electrical tuning of reflection phase over 180° at 1.55 um and 5.94 um, respectively (see Figure 1(d)) [27]. Very recently, Atwater and coworkers introduced a dual-gated field-tunable MIM metasurface that enables ultrawide reflection phase modulation exceeding >300° at the wavelength of 1.55 um. Such dual-gated ETM consists of an Al background plate, a gated-dielectric/ITO/gated-dielectric heterostructure and a periodic array of Al meta-atoms with “fishbone” pattern, as shown in Figure 1(e). It features two-charge depletion/accumulation at both top and bottom ITO/gated-dielectric interfaces, facilitating a large variation of the complex refractive index of the ITO layer via carrier density modulation at both interfaces and leading to such wide phase tuning range [28]. Very recently, Forouzmand et al. proposed ITO-integrated all dielectric tunable metasurfaces that can realize relatively large phase modulation in both transmission and reflection modes at two different wavelengths in NIR regime [82]. In addition, many other TCO materials (e.g., Al:ZnO; Ga:ZnO) exhibiting ENZ properties have also been widely used to realize ETMs in the NIR regime [83].

Most ETMs introduced above are based on carrier-density modifications inside given materials, which result in changes in both real and imaginary parts of their permittivity. While changing Im(*ε*) can indeed help in making tunable metadevices (say, in [28, 60]), it also introduces inevitable Ohmic losses which can degrade the performances of realized devices. It is highly desired to find a mechanism that* only* modulates the real part of permittivity. Liquid crystals (LCs), formed by elongated molecules with orientations controlled by external electric fields, exhibit electrically tunable Re(*ε*). Incorporating LCs into MTM designs, one can realize ESTs with resonant properties strongly modulated via applying electric field across the LCs. Such a mechanism works in a wide working frequency range covering THz to visible regime [29, 84–86], achieving various functional devices, such as spatial light modulators (SLMs) [29, 84], color tuning [85], and tunable wave-plate [86]. For instance, in 2013, Padilla and coworkers experimentally demonstrated a tunable absorber in the THz regime based on an MIM structure with an LC layer as the dielectric spacer. A bias voltage applied between the ground plane and the upper resonator can change the refractive index of involved LC layer by an amount of 0.19, resulting in a 30% absorption modulation at 2.62 THz associated with a resonant frequency shift of ~4% [29], as shown in Figure 1(f). They further improved the performance of such LCs based tunable absorber design to THz SLMs with experimentally achieved modulation depth of 75% [84]. Pixelating these tunable meta-atoms, one can realize different types of tunable devices such as SLMs, sensors, and imagers, which usually do not need extremely high modulation speed (limited to 1 KHz).

### 2.2. Mechanically Switchable Metasurfaces (MSMs)

The tuning mechanisms described in last subsection are mainly based on modifying the optical environments (i.e., substrates and spacers) of metallic resonators. Alternatively, one may realize dynamically reconfigurable metadevices through mechanically changing the geometrical structures of constitutional meta-atoms or altering the distances between adjacent meta-atoms or that between meta-atoms and their substrate. The latter can manipulate the near-field interactions between meta-atoms, enabling dramatic modifications on the EM properties of the whole metasystem.

**Figure 2:**
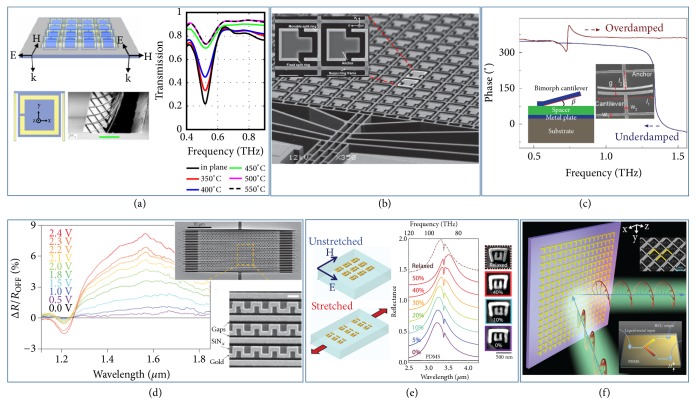
*Mechanically switchable metasurfaces.* (a) Schematics of a THz thermomechanical tunable metasurface with meta-atom design and measured transmission spectra at different temperatures. (b) A micromachined MSM composed by reconfigurable asymmetric SRRs for controlling THz waves. (c) MEMS-controlled phase transitions between underdamped and overdamped resonators in a THz MSM. Insets: schematics and photos of the realized MSM. (d) A MSM realized with NEMS technology for actively controlling the transmission/reflection properties of near-IR light. Inset: image of the fabricated metadevice with detailed meta-atom structure. (e) Dynamic resonant-frequency tuning achieved with a MSM based on a flexible substrate in IR regime. (f) Active polarization conversion of microwaves achieved by a liquid-metal-incorporated metasurface realized with microfluidics technology. Insets: schematics of the designed tunable meta-atom (bottom panel) and a zoomed-in view of the microfluidic metasurface with galinstan filled-in (up-panel). Reproduced with permissions: (a) from [30] ©2009 APS; (b) from [31] ©2011 Wiley-VCH; (c) from [32] ©2017 Wiley-VCH; (d) from [33] ©2013 NPG; (e) from [34] ©2010 ACS; (f) from [35] ©2017 Wiley-VCH.

Micro-electro-mechanical-systems (MEMS) and nano-electro-mechanical-systems (NEMS) are two widely used technologies to realize MSMs in different frequency domains for different applications [30–33, 87–91]. An early MSM was reported in 2006, which is a tunable microwave band-stop filter where micromachined switches were incorporated inside an EM bandgap structure [87]. In 2009, Tao* et al. *realized a THz thermomechanical switchable metasurface, containing an array of SRRs patterned on bimaterials cantilevers. As shown in Figure 2(a), the cantilevers can be bend upwards out of plane with external thermal stimuli, resulting in a remarkable tuning of the electric and magnetic responses of such metasurface (e.g., a ~50% modulation on transmission at 0.5 THz achieved by synchronously reorienting the SRRs within their unit-cells) [30]. Several other MSMs were subsequently demonstrated in both THz and optical regimes, based on electrically activated MEMS switches [31, 32, 89, 90]. In one typical type of designs, the devices consist of arrays of carefully designed meta-atoms, each of which exhibits two separated parts, one fixed on the substrate while the other patterned on a floating frame which can be driven by actuators [31, 89]. Adjusting the relative positions between these two portions can deform the lattice structure and, in turn, change the near-field interactions between adjacent meta-atoms, thus allowing for efficient tailoring on EM properties of the whole devices. For instance, Zhu* et al*. fabricated an electrical MSM device based on the above-mentioned techniques, adopting an asymmetric SRR (Figure 2(b)) as their basic meta-atom [31]. The authors experimentally demonstrated a large relative switch (up to 31%) on resonant frequency with a response time of 500 *μ*s at ~2 THz, suggesting the application potentials of the proposed device in THz polarization-switch and imaging. In another class of systems, researchers chose to utilize external-stimuli-sensitive flexible MEMS cantilevers to construct MSMs [32, 90]. For example, Cong* et al*. experimentally demonstrated a wide-range amplitude and phase modulation on THz waves in an MEMS-based metadevice (Figure 2(c)) [32]. The working mechanism is based on the underdamped-to-overdamped resonator transition established in Ref. [26], which in this case is driven by varying the system's radiative Q factor via electrically controlling the gap distance between cantilevers and spacers. Moreover, NEMS technology has also been adopted to achieve electrical MSMs in IR regime [33, 91]. A reconfigurable metasurface, driven by electrostatic forces, was reported in 2013, which consists of a metallic “meander-near-the-wire” pattern manufactured on a grid of flexible dielectric membrane (Figure 2(d)) [33]. A continuous reflection modulation (up to 8%) on incident light (at 1550 nm) with a mega-hertz modulation rate was experimentally achieved by changing the configuration of the metastructure through NEMS technology.

Flexible substrates offer another straightforward way to realize reconfigurable metadevices [34, 92–96]. In 2010, Pryce* et al.* experimentally demonstrated wide-range tunability on working wavelength (∆*λ*~ 400 nm), based on a reconfigurable metasurface composed by planar, coupled SRR arrays adhered to an elastic polydimethylsiloxane (PDMS) substrate. As shown in Figure 2(e), mechanical deformation can stretch the substrate and, in turn, enlarges the interdistances between metallic resonators. As a result, both inter-SRR couplings and the capacitance contributed by the SRR gap are modulated accordingly, leading to a large resonant-frequency tuning [34]. Recently, Halas's group reported a plasmonic metadevice integrating a square array of aluminum nanoantennas with an elastomeric substrate. They experimentally demonstrated that the metadevice exhibits continuously tunable EM responses covering the entire visible spectrum, as well as an active image switching, enabled by mechanically stretching the structure [94]. Despite the limited modulation speed of MSMs based on elastic materials, one can realize different functional devices in different frequency ranges, such as switchable metaholograms and tunable metalenses.

Microfluidics is another powerful technology to achieve MSMs [35, 97, 98]. To achieve the meta-atoms of tunable EM responses, people have designed an array of cavity that can be filled with liquid metal or solvent in a controllable manner based on microfluidics technology and pneumatic valves. For example, Wu* et al.* experimentally demonstrated a microwave active polarization converter/attenuator with galinstan-based metasurfaces (see Figure 2(f)). By actively controlling the arm length of the L-shaped galinstan resonators via microfluidic channels, the authors demonstrated that the polarization of reflected EM wave can be actively switched among different states, such as linear, circular, and elliptic polarizations, within a broad frequency band (60% relative working bandwidth) and a large incident angle range of 45° [35]. However, the limited modulation speed (up to 100 Hz) and the difficulty on pixelated control for high-frequency EM waves have restricted its potential for versatile applications. 

### 2.3. Optically Tunable Metasurfaces (OTMs)

**Figure 3:**
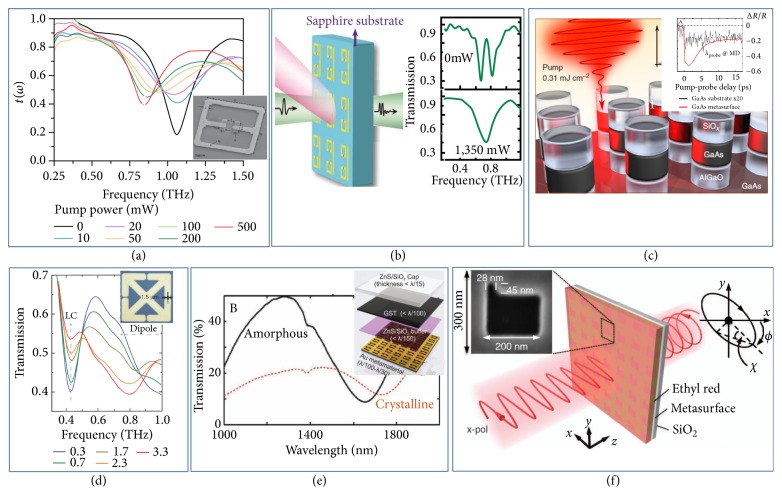
*Optically tunable metasurfaces. *(a) A semiconductor-based OTM achieving dynamical modulation on transmitted THz wave under different pumping conditions. Inset: SEM picture of the involved meta-atom. (b) Optically controlled EIT peak tuning achieved in a THz OTM with photoconductive silicon incorporated. (c) Illustration of ultrafast all-optical tuning of Mie-resonant mode in a GaAs involved dielectric OTM. Inset: measured transient reflectance of the metadevice and the substrate under the same optical pumping. (d) Measured field-dependent nonlinear transmission spectra of a THz OTM with VO_2_ incorporated. Inset: optical image of the SRR deposited on a VO_2_/sapphire substrate. (e) All-optical and high-contrast transmission switching of near-IR light in a metadevice incorporated GST nanolayer. Inset: schematics of the multilayer structure of the designed OTM. (f) An OTM realized with optically sensitive polymers for dynamic polarization manipulation of visible light. Reproduced with permissions: (a) from [36] ©2009 NGP; (b) from [37]; (c) from [38]; (d) from [39] ©2012 NGP; (e) from [40] © 2013 Wiley-VCH; (f) from [41] © 2017 NPG.

Optical modulation through ultrafast light pulses is so far the fastest modulation technology, which provides another way to realize active metasurfaces typically constructed with meta-atoms involving optically sensitive materials. Semiconductors belong to such kind of materials whose conduction carriers (and in turn, dielectric constants) strongly depend on external optical pumping, and therefore, they are widely used to realize active metadevices at frequencies ranging from THz to the visible [36, 37, 99, 100]. The first OTM was reported in 2006 by Padilla* et al*., who patterned a copper SRR array on a semi-insulating gallium arsenide (GaAs) substrate [99]. The authors experimentally demonstrated that, under ultrafast (50 fs pulse width) light pumping at 800nm, photoexcitation induced conductivity in GaAs can short out the gaps of metallic SRRs, thereby tuning off the electric resonance of the metastructure, yielding a strong modulation on transmission amplitude at 0.56 THz. Several years later, instead of using semiconductors as substrates, Padilla and coworkers realized another OTM by directly combining metallic and semiconducting materials to form their composite meta-atom (inset to Figure 3(a)) [36]. As shown in Figure 3(a), the designed composite meta-atom, placed on a silicon-on-sapphire substrate, consists of a metallic SRR with central gap filled with photoconductive silicon. Such configuration permits an optically stimulated capacitance change of the SRR gap, thus leading to a significant resonant frequency shift upon pumping. With an optical pump fluence of 0.5 mj/cm^2^, the authors observed a 20% resonant-frequency-tuning of the device at ~0.9 THz. Based on similar configuration, Gu* et al.* designed and fabricated a THz OTM to achieve optically controlled electromagnetically-induced transparency (EIT) associated with actively tuned EM wave group delay. As shown in Figure 3(b), the meta-atom consists of a metallic cut wire and two SRRs with gaps filled with photoconductive silicon [37]. Pumped by light at 800 nm with a power 1350 mW, the whole device undergoes an on-to-off EIT peak modulation, with transmission amplitude varying from 85% to 43% accompanied with a 5.74 ps change in EM wave group delay, at the frequency of 0.74 THz.

On the other hand, high-index semiconducting materials can be directly used to build functional meta-atoms based on the Mie mechanism [101]. Therefore, optically pumping such all-dielectric meta-atoms can efficiently modulate the EM responses of the constructed device, through changing the dielectric constant of the constitutional semiconducting material. Figure 3(c) presents such an OTM, formed by Silica/GaAs/AlGaO nanopillars grown on a bulk GaAs substrate. By means of fs pump-probe spectroscopy, Shcherbakov* et al.* demonstrated that the absolute reflection amplitude from the metasurface can be modulated by 35%, under a low pump fluence and with a recovery time of ~6 ps. The physical origin comes from ultrafast tuning of spectral position of the magnetic dipole resonance (up to ∆*λ*=30 nm), induced by refraction-index change of GaAs under external optical pumping [38]. Such a metaconfiguration could work at both visible and NIR regimes, with appropriately chosen semiconducting materials.

Light-induced phase-change materials (PCMs) [39–42, 102–107] were another class of candidates to realize OTMs. In 2012, D. Averitt and coworkers experimentally demonstrated a dynamical modulation on THz transmission (with modulation depth of ~38% at 0.41 THz) through a metasurface with vanadium dioxide (VO_2_) incorporated (see Figure 3(d)). The building meta-atom of such an OTM is a gold SRR with a 1.5 *μ*m gap, patterned on a thin VO_2_ film deposited on a sapphire substrate. Such optical modulation arises from the insulator-metal transition of the VO_2_ film induced by high-field THz pulses (3.3MV cm^−1^), aided by strong subwavelength field enhancement generated by the SRR [39]. Actually, VO_2_ has been popularly used for constructing active metasurfaces, since its insulator-metal transition can be induced by different probing (e.g., optical, electrical, and thermal ones) [42, 104]. Germanium-antimony-tellurium (GST) is another PCM [22, 40, 105], which exhibits two phases (crystalline and amorphous phases) with distinct optical properties (i.e., dielectric constants) under different conditions. Phase changes in GST materials (with optical contrast, high tuning speed, and good thermal stability) can be induced by different external stimuli (e.g., electrical, optical, and thermal ones), which makes GST an ideal material to build tunable metasurfaces [40, 106, 107]. For instance, in 2013, Zheludev and coworkers demonstrated an all-optical OTM through hybridizing GST with a metallic metasurface consisting of asymmetric split ring slots in a metal film (see Figure 3(e)). Using a high-intensity pulsed light (at 660 nm) to induce the amorphous-crystalline phase transition in GST, the authors experimentally observed an obvious shift in resonant frequency of the metasurface, resulting in a 400% modulation on absolute reflection amplitude from the device, at the wavelength around 1550 nm [40].

In addition, some optically sensitive molecules or polymers exhibiting binary isomeric states can also be good candidates to design OTMs. Very recently, Ren* et al.* reported an OTM in optical regime, which can actively modulate the polarization characteristics of transmitted light beam upon optical stimulation. The realized OTM combines a plasmonic metasurface, which is a metal film drilled with an array of L-shaped slits placed on a fused quartz substrate, and a thin layer of photoisomerizable azo-ethyl-red. The tunability of such configuration comes from the strong coupling between plasmonic resonances of the meta-atoms and the switchable isomeric state of ethyl-red polymer. As shown in Figure 3(f), the authors experimentally demonstrated a >20° nonlinear optical polarization modulation at wavelengths around 820 nm, under weak tuning light excitation of just a few mille-watts [41].

### 2.4. Thermally Tunable Metasurfaces (TTMs)

**Figure 4:**
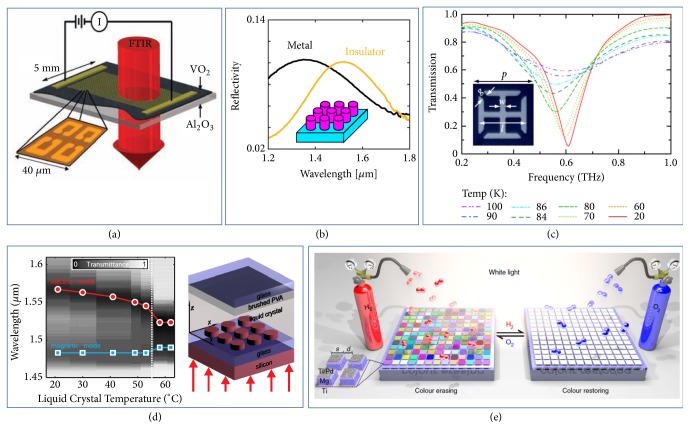
*Thermally and chemically switchable metasurfaces.* (a) A VO_2_–hybridized TSM for actively controlling THz waves. (b) A near-IR TSM constructed with VO_2_ resonators and its temperature-controlled optical spectra. (c) A superconductor-based TTM for transmission modulations on THz wave. Inset: image of the fabricated SRR meta-atom. (d) A TSM combining a dielectric metasurface (silicon nanodisk array) and a layer of LCs to achieve transmission modulation on near-IR light. (e) A chemically switchable metadevice formed by a plasmonic metasurface composed of hydrogen-responsive Mg nanoparticles. Reproduced with permissions: (a) from [42] ©2009AAAS; (b) from [43] ©2018ACS; (c) from [44] ©2010 APS; (d) from [45] ©2015ACS; (e) from [46].

Combining passive metasurfaces with thermally sensitive materials (e.g., PCMs, LCs, and superconductors), one can realize thermally tunable metasurfaces (TTMs). In last subsection, we have mentioned that VO_2_ is a PCM exhibiting a metal-insulator transition under certain conditions. Such phase transition with lower transition temperature compared to GST can be induced not only by light pumping, but also by varying the ambient temperature. As a result, VO2 has been widely used to design TTMs in different frequency regimes [42, 108, 109]. For instance, in 2009, Driscoll* et al*. realized a TTM composed by a metallic SRR array placed on a VO_2_ substrate and experimentally demonstrated 20% tuning on THz transmission through it (see Figure 4(a)) via changing temperature. The authors further proposed a memory device based on such a TTM [42]. Very recently, Butakov* et al.* constructed a TTM consisting of an array of wire-and-disk resonators put on a VO_2_ layer and experimentally demonstrated that dramatic thermal tuning on reflectance induced resonance-frequency shift trigged by the insulator-metal transition of the VO_2_ substrate (Figure 4(b)) [43]. In addition, Chalcogenide phase change materials such as GST are also widely used to realize various switchable functionalities at different working frequencies with thermal stimuli, such as spatial light modulation [105], beam steering, and bifocal lensing [106].

Superconductor is an intriguing material to design TTMs in microwave and THz regimes, since its conductivity strongly depends on temperature which may trigger the transition from superconducting phase to normal one [44, 110]. In 2010, Chen* et al*. fabricated a TTM based on high-temperature superconducting materials and experimentally demonstrated that its resonance frequency can be tuned efficiently via changing temperature. The realized TTM consists of electric SRRs, made by YBCO superconducting thin films, deposited on a LaAlO3 substrate. As shown in Figure 4(c), the strength of electrical resonance of the realized TTM decreases as increasing the ambient temperature, manifested by the much broadened and weakened transmission dip [44]. In a parallel line, Jin* et al.* reported that a superconducting THz metasurface, consisting of double SRRs made by niobium film, exhibits an actively tunable EM response stimulated by varying temperature or applying magnetic field [110].

Thanks to their temperature-dependent refraction indexes, LCs have also been widely used to realize TTMs at different frequency ranges, such as spatial light modulators, active polarization controllers [45], and beam deflector [111]. Recently, Sautter et al. fabricated a TTM through combining nematic LCs with a dielectric metasurface consisting of silicon nanodisk array. They experimentally demonstrated that the TTM exhibits a thermally controlled resonant frequency shift (up to 40 nm) with a huge transmission contrast (up to 500%) in telecom wavelength regime [45], as shown in Figure 4(d).

### 2.5. Chemical Approaches to Realize Tunable/Reconfigurable Metasurfaces

Using chemical approach to alter the chemical components of constituent materials forming a metasurface, one can change the EM properties of a whole device accordingly, thereby achieving the desired optical tunability. Recently, Duan* et al.* experimentally demonstrated the ability to reversibly control the hydrogenation and dehydrogenation of magnesium (Mg) nanoantennas (see Figure 4(e)), enabling a reversible switch between metallic (Mg) and insulating (MgH_2_) states of the constitutional material forming the nanoantennas, eventually leading to a significant resonant frequency shift of the meta-atom in the optical regime [46]. Such a smart material-processing technology can be used to realize dynamically controlled plasmonic color displays and hydrogen detections.

## 3. Inhomogeneous Tunable/Reconfigurable Metasurfaces

In previous section, we have summarized available approaches to realize* homogeneous* tunable/reconfigurable metadevices with constitutional meta-atoms tuned* uniformly*. However, more fascinating effects would be achieved in* inhomogeneous* active metasurfaces with meta-atoms tuned* independently*, such as beam steering, programmable imaging, and holograms. In principle, one can implement the tuning schemes developed in Section 2 to independently tune the EM response of each individual meta-atom in an inhomogeneous metasurface. However, grand challenges exist in reality, especially for constructing metadevices working at high frequencies, caused by issues arising from both scientific and engineering aspects. As a result, naturally, progress along this direction is much less than that along the homogeneous active metasurfaces. In this section, we will briefly review recent efforts devoted to this subfield, including achievements and challenges.

**Figure 5:**
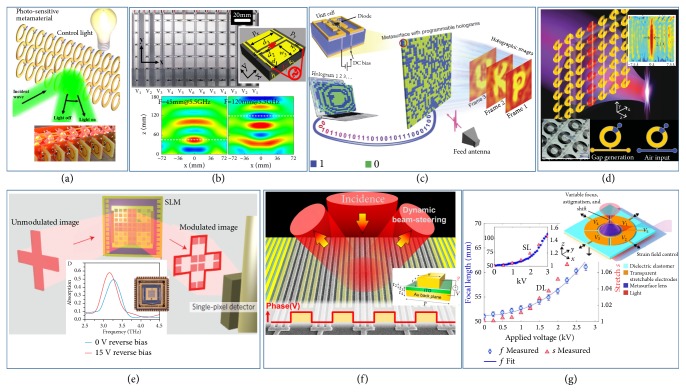
*Inhomogeneous tunable metasurfaces.* (a) A tunable metadevice formed by meta-atoms incorporated with photosensitive materials to achieve light-controllable reflections on incident microwaves. Inset: picture of the fabricated sample. (b) Aberration-free and functionality-switchable microwave metalenses with individual meta-atoms controlled with different applied bias voltages. (c) A microwave coding-metasurface achieving reprogrammable holograms controlled by voltages applied on PIN diodes embedded in constitutional meta-atoms. (d) A microwave tunable flat lens realized with random-access reconfigurable metamaterial based on microfluidic technology. Insets: picture and schematics of the microcavity filled with liquid metal. (e) Schematics of the single-pixel imaging process realized with a THz Spatial light modulator (SLM). Insets: photo of the fabricated SLM and measured absorption spectra of a single pixel under two bias voltages. (f) Gate-controlled diffractions of light at a MIM metadevice with ITO as its spacer, under appropriately chosen electric gating. (g) An adaptive metalens formed by dielectric metalens and artificial muscles to realize simultaneous controls on focal length, astigmatism, and shift of the formed image, enabled through electrical-strain control on the artificial muscles. Reproduced with permissions: (a) from [47] © 2012 APS; (b) from [48] © 2016 AIP. (c) from [49]; (d) from [50] © 2015 WILEY-VCH; (e) from [51] © 2014 Macmillan Publishers Limited; (f) from [52] ©2016 ACS; (g) from [53].

In the microwave regime, voltage-driven elements, such as diodes/varactors/transistors, have been widely used to design active meta-atoms achieving dynamically tunable EM responses, thanks to their miniaturized sizes compatible with passive metallic resonators. Since each meta-atom can be independently controlled by the tunable element embedded, one can assemble these meta-atoms to form an* inhomogeneous* metasurface with phase/amplitude profiles accurately determined by the voltages applied on different meta-atoms, thus offering the metadevice desired dynamical wave-manipulation functionalities. More exciting progress has been made using such a scheme [47–49, 112–117]. In 2003, Sievenpiper* et al*. experimentally demonstrated an electrically steerable reflector in the microwave regime by incorporating varactor diodes into a reflective array in MIM configuration. By programming the reflective phase gradient on the device, the authors demonstrated that the reflected microwave beam can be electrically steered over +/- 40° for both polarizations [112]. Recently, Shadrivov* et al.* proposed a microwave light-tunable metadevice composed by an array of broadside-coupled SRRs, each containing a varactor diode connected with a light emitting diode (LED) [47]. Manipulating the field pattern illuminated on the LED array through controlling the external light radiation, one can individually control the voltages supplied to those varactor diodes, which, in turn, dynamically modulate the EM functionality of whole device. Based on this strategy, the authors successfully realized active switch between focusing and defocusing functionality for the fabricated metadevice (see Figure 5(a)). Very recently, Zhang et al. proposed and realized remote-mode digital coding metasurfaces composed of light-addressable meta-atoms, which enable actively reconfigurable radiation beams in microwave regime by adjusting the intensity of light-emitting diode light [118]. In a parallel line, Xu* et al.* proposed a “tunable” approach to solve the chromatic issue in passive metasystems constructed by resonant meta-atoms exhibiting Lorentz-shape responses. The idea is to precisely rectify the dispersion-induced phase distortions in different meta-atoms at frequencies away from the target one, through changing the bias voltages applied on the varactor diodes incorporated in those meta-atoms. Based on this concept, the authors experimentally demonstrated a tunable microwave gradient metasurface functioning as a single-mode high-efficiency anomalous reflector at all frequencies within a wide band [113] and a metadevice exhibiting dynamically switchable functionality between specular reflector and surface-wave convertor [113]. Moreover, the authors further fabricated a tunable microwave metalens (Figure 5(b)) that can either exhibit aberration-free focusing functionality within a wide frequency band or exhibit dynamically switchable focusing performances at a fixed frequency, depending on how the gating voltages are applied [48]. Very recently, Cui's group introduced the concept of reprogrammable coding metasurfaces to achieve dynamically controlled manipulations on microwaves [49, 115, 116]. The adopted meta-atom is a MIM structure with top resonator loaded with a varactor diode. Controlling the bias voltage applied across the diode can yield two different reflection phases for the meta-atom, i.e., 0 and *π*, which are defined by “0” and “1” states, respectively. Therefore, by reprogramming the bias voltages applied on these meta-atoms, the authors were able to control the phase distribution encoded on the whole metadevice, thereby creating multiple holographic images that can be dynamically switched in real time (Figure 5(c)) [49].

The active metadevices mentioned above are all working in reflection geometry, which are relatively easy to realize but are sometimes unfavorable for certain applications. To realize tunable/reconfigurable metadevices in transmission mode, one needs to precisely control both phase and amplitude of locally transmitted wave through each meta-atom, which is quite challenging. Recently, Chen* et al.* experimentally demonstrated a microwave reconfigurable metalens based on a tunable Huygens' surface, which can simultaneously control multiple focal spots at distinct spatial positions. Such an active metadevice was constructed by a 2D array of composite meta-atoms exhibiting both electric and magnetic responses; inside each a voltage-controlled varactor is embedded. Again, varying the voltages applied across these varactors, the authors can precisely and dynamically control the distribution of transmission phase on the metasurface, yielding a dynamical manipulation on the field pattern generated at the transmission side. The measured absolute focusing efficiency is about 36% due to material losses and deviations in phase/amplitude distributions, and the modulation speed reaches 100000 or 105 switches/s [114]. Meanwhile, microfluidics technology was also widely used to realize tunable metadevices in the microwave regime, such as tunable metalenses [50], dynamically controlled beam deflectors [119], and tunable polarization convertors [35, 120]. The working mechanism is that microfluidic network can individually and continuously control the EM response of each meta-atom, through varying the filling factor of metal liquid or solvent. For instance, in 2015, Liu's group constructed a reconfigurable metalens, formed by casting resonators inside microfluidic channels, and then experimentally demonstrated a dynamically switchable focusing effect based on such a metalens, as shown in Figure 5(d) [50].

However, these technologies are difficult to implement at frequencies higher than GHz, due to lacking suitable voltage-driven miniaturized elements working at those frequencies. Along with rapid development on nanofabrication and material sciences, enormous efforts have been devoted to exploring new tuning technologies in both THz and optical regimes. In 2014, Watts* et al.* experimentally demonstrated a THz spatial light modulator (SLM) for high-frame-rate and high-fidelity THz imaging with a single-pixel detector (see Figure 5(e)). Such THz SLM was constructed by 8x8 pixels of polarization-sensitive and electrically tunable meta-absorbers in MIM configuration and could function as a real-time tunable and spectrally-dependent spatial mask. The EM property of each pixel/meta-absorber can be individually and dynamically controlled by varying the bias voltage applied across the spacer layer (an n-doped GaAs layer) of the MIM meta-atom [51]. Recently, Atwater's group experimentally demonstrated an electrically addressable phase (184°) and amplitude (30%) modulation on light reflected light from MIM metagrating in near-IR regime. By varying the bias voltage applied across the spacer layer (a transparent conducting oxide (TCO) layer) of the MIM metagrating, the authors can dynamically tune the reflection phase of the whole device, thanks to the field-modulated permittivity of TCO operating at the ENZ condition. The working principle of such a device is essentially the same as that of the graphene metasurface, based on the same underdamped-to-overdamped resonator transition. What is more, the authors further employed such tunable phase shift to design electrically controlled 2-level phase-grating and experimentally demonstrated that the ±1 order diffracted beams angle can be actively steered by gating metagratings with different periodicities, as shown in Figure 5(f) [52]. Recently, stretchable substrates have been widely used to realize inhomogeneous tunable/reconfigurable metadevices in the optical regime, such as tunable color generator [93] and tunable metalens [53, 94]. In 2018, Capasso's group experimentally demonstrated electrically switchable large-area metalenses controlled by artificial muscles (dielectric elastomer actuators), which are capable to perform simultaneously focal length tuning, astigmatism, and image shift corrections [53]. The metalens's phase profile can be coupled to voltage-induced stretching by bonding itself with artificial muscles. As shown in Figure 5(g), such an electrically adaptive metalens (with single-layer configuration) exhibits 107% focal length modulation at 1550 nm.

## 4. Conclusions and Perspectives

In conclusion, we have briefly summarized recent exciting progress on tunable/reconfigurable metasurfaces with meta-atoms controlled both uniformly and individually by different types of external knobs, focusing particularly on available tuning mechanisms and potential applications of realized metadevices. These research outputs have offered metasurfaces significantly expanded capabilities to control EM waves in different frequency regimes, providing a promising platform to realize functional devices meeting different applications, such as SLMs [33, 36, 121, 122], switchable color filter [123], tunable metalenses [53, 96, 124], and polarization controller [31, 41]. However, although tunable metadevices can bring expanded controllability on EM waves, they inevitably exhibit certain disadvantage as compared to passive ones. For example, active metasurfaces usually possess more complex configurations than their passive counterparts, since active materials and external control systems must be integrated into the systems. Also, active metadevices may exhibit degraded working efficiencies due to the absorption of added tunable materials.

Overall, it is worth providing a clear comparison on different tuning approaches to realize tunable and reconfigurable metadevices at different working frequency ranges with different functionalities (see Table 1). One of the commonly used approaches to tune EM properties of metadevices is through the free carrier doping in conductive materials with electrical gating and photoexcitation methods. Conventional semiconductors (e.g., GaAs, Silicon, and germanium), atomically thin 2D materials (e.g., graphene; MoS2), and transparent conducting oxides or nitrides (e.g., ITO; AZO) are widely used in the spectral range covering THz and up to visible regime. The tuning approaches based on phase transition under different external knobs (e.g., electrical, optical, and thermal stimuli) have played important roles in active metasurfaces, thanks to the high tunability in refractive index of the PCM (e.g., GST, LCs, VO2, and superconductor) in the spectral range from GHz to visible. Mechanical tuning offers another effective way to switch the EM properties of metadevices by reconfiguring the shape and surrounding environment of meta-atom by the means of MEMS/NEMS, elastic subtract, or microfluidics. Another notable approach to realize tunable metadevices below and up to GHz regime is through electrical control on diodes/varactor/transistor hybridized with metasystems. Due to the restriction on article length, we could not cover more important progress in this rapidly growing subarea, such as spatiotemporal metasurfaces [15, 125–127], active metasurfaces for other waves including acoustic [128–132] and thematic waves [133, 134].

Although we have witnessed the great progress achieved in the field of tunable and reconfigurable metasurfaces, several grand challenges still exist. For example, to freely control EM waves dynamically, one needs to manipulate both phase and amplitude of local EM field* independently*. However, many currently existing mechanisms modulate the phases and amplitudes of EM waves* simultaneously* and in a* locked* way, which significantly limits their applications in realizing functional metadevices. Therefore, it is highly desired to find new mechanisms that can achieve* wide-range* yet* decoupled* modulations on EM phase and amplitude, particularly, at high frequencies. Meanwhile, establishing new technologies to realize* local* controls on light waves at optical frequencies is another major challenge in this subfield, since many techniques developed in GHz/THz regimes do not work at high frequencies, due to significantly increased difficulties in fabrications and material processing. The third grand challenge arises from material aspect. So far, most realized tunable/reconfigurable metadevices are based on plasmonic systems, which are incompatible with current CMOS technology. Such an issue could be an obstacle for integrating the developed metadevices with existing photonic systems. Fortunately, emergence of dielectric metasurfaces and new materials (e.g., transition metal nitrides and transparent conductive oxides) provides new possibilities to achieve COMS-compatible tunable metadevices. In our opinion, these grand challenges actually provide new opportunities for scientists working in this field, which can eventually push the field forward.

Before concluding this review, we would like to mention several important future directions in this subfield, based on our perspectives:

[1] Spatiotemporal tunable metasurfaces. Recently, as a new branch of active metasurface research, spatiotemporal metasurfaces, incorporating both spatial- and time-varying gradients of abrupt phases, have attracted rapidly growing interests, due to the stimulated new physical effects not presented in their static counterparts [15, 18]. For example, the time-varying gradient can impart additional frequency to the incoming EM wave, thereby resulting in a Doppler-like shift in the frequency. Shalaev and coworkers showed that spatiotemporal metasurfaces can lead to a generalized Snell's law, where both momentum and energy conservation are relaxed and several fascinating applications could be achieved [127]. Cui et al. experimentally demonstrated that the harmonics of the scattering wave can be freely manipulated in both amplitude and phase with time-varying metasurfaces, by controlling the time-sequences of the external sources [125, 126]. We expect more fascinating discoveries to appear in this subfield.

[2] Active metadevices for the waves other than EM wave. Since the concept of metasurfaces/metamaterials can be extended to manipulate waves other than EM wave (e.g., acoustic wave [128, 129, 131, 132] and thematic wave [133, 134]), many research efforts have been devoted in the new subfield of tunable acoustic and thematic metamaterials/metasurfaces, leading to many fancy physical effect and practical applications [133, 134]. For example, Li et al. theoretically and experimentally demonstrated that robust and switchable acoustic asymmetric transmission can be achieved through gradient-index metasurfaces by harnessing carefully tailored losses [129]. Huang and coworkers established an approach to design switchable thermal cloaking and experimentally realized a macroscopic thermal diode of huge potential applications related to heat preservation and dissipation [133].

[3] Tunable metasystem for real applications. Facing a different application scenario, different tunable and reconfigurable metadevices can be implemented based on different structure with different tuning mechanisms and active materials. However for real applications, lots of efforts are still needed, for example, in integrating tunable metasurfaces with conventional electrooptical devices, in large area low-cost fabrications, and in constructing software-defined platforms for automatic control on metadevices.

Novel ideas in the context of tunable and reconfigurable metasurfaces, beyond those introduced in this review, will surely arise in the near future, leading to versatile and powerful applications, such as tunable lenses, laser beam steering, and 3D dynamic holographic display, to name just a few. Based on the progress over the past decade, we can surely have a rosy prospective on the future of tunable and reconfigurable metasurfaces.

## Figures and Tables

**Table 1:** Comparison of different approaches to realize tunable and reconfigurable metadevices.

Tuning Approach	Materials	External knots	Operation spectrum	Functionalities	Modulation range/speed	Ref.
Carrier doping	Semiconductors	Electrical	THz to Visible	modulator	55%/2MHz	[58]
		Optical			90%/NA	[38]
	Graphene	Electrical	THz to NIR	polarizer/absorber	243°/NA	[26]
					100%/20GHz	[77]
	TCO	Electrical	NIR to Visible	phase modulator	30%/10MHz	[52]

Phase transition	Liquid Crystals	Electrical	GHz to Visible	modulator/beam deflector/color filter	70%/NA	[84]
		Thermal			12°/NA	[111]
		Optical			18%/NA	[123]
	VO_2_	Electrical	THz to visible	modulator	80%/1KHz	[104]
		Thermal		modulator	20%/NA	[42]
		Optical		modulator	38%/NA	[39]
	GST	Electrical	THz to Visible	color tuning	100%/NA	[107]
		Thermal		beam switching	NA	[106]
		Optical		modulator	400%/10MHz	[40]
	Superconductor	Thermal	GHz to THz	modulator	35%/NA	[44]

Mechanics	MEMS/NEMS	Electromechanical	GHz to Visible	modulator	31%/1KHz	[31]
					8%/ to MHz	[33]
	Elasticity	Mechanical	GHz to Visible	Color tuning/Tunable lens	27%/NA	[94]
					NA/NA	[53]
	Microfluidics	Microfluidics	GHz to Visible	polarization converter/color printing	NA/NA	[35]
					NA/62Hz	[98]

Capacitance	Varactor/PIN diode	Electrical	MHz-GHz	Tunable lens/reprogrammable hologram	NA/NA	[48]
					NA/100KHz	[49]

## References

[1] Liu Y., Zhang X. (2011). Metamaterials: a new frontier of science and technology. *Chemical Society Reviews*.

[2] Engheta N., Ziolkowski R. W. (2006). *Metamaterials: Physics and Engineering Explorations*.

[3] Soukoulis C. M., Wegener M. (2011). Past achievements and future challenges in the development of three-dimensional photonic metamaterials. *Nature Photonics*.

[4] Ding F., Pors A., Bozhevolnyi S. I. (2017). Gradient metasurfaces: a review of fundamentals and applications. *Reports on Progress in Physics*.

[5] Yu N., Genevet P., Kats M. A. (2011). Light propagation with phase discontinuities: Generalized laws of reflection and refraction. *Science*.

[6] Sun S. L., He Q., Xiao S. Y., Xu Q., Li X., Zhou L. (2012). Gradient-index meta-surfaces as a bridge linking propagating waves and surface waves. *Nature Materials*.

[7] Yu N., Capasso F. (2014). Flat optics with designer metasurfaces. *Nature Materials*.

[8] He Q., Sun S., Xiao S., Zhou L. (2018). High-efficiency metasurfaces: principles, realizations, and applications. *Advanced Optical Materials*.

[9] Chen H.-T., Taylor A. J., Yu N. (2016). A review of metasurfaces: physics and applications. *Reports on Progress in Physics*.

[10] Hsiao H.-H., Chu C. H., Tsai D. P. (2017). Fundamentals and applications of metasurfaces. *Small Methods*.

[11] Kruk S., Kivshar Y. (2017). Functional meta-optics and nanophotonics governed by mie resonances. *ACS Photonics*.

[12] Decker M., Staude I. (2016). Resonant dielectric nanostructures: A low-loss platform for functional nanophotonics. *Journal of Optics*.

[13] Zheludev N. I., Kivshar Y. S. (2012). From metamaterials to metadevices. *Nature Materials*.

[14] Fan K., Padilla W. J. (2015). Dynamic electromagnetic metamaterials. *Materials Today*.

[15] Shaltout A. M., Shalaev V. M., Brongersma M. L. (2019). Spatiotemporal light control with active metasurfaces. *Science*.

[16] Shaltout A. M., Kinsey N., Kim J. (2016). Development of optical metasurfaces: emerging concepts and new materials. *Proceedings of the IEEE*.

[17] Padilla W. J., Averitt R. D. (2017). Properties of dynamical electromagnetic metamaterials. *J. Opt*.

[18] Shaltout A. M., Kildishev A. V., Shalaev V. M. (2016). Evolution of photonic metasurfaces: From static to dynamic. *Journal of the Optical Society of America B: Optical Physics*.

[19] Li A., Luo Z., Wakatsuchi H., Kim S., Sievenpiper D. F. (2017). Nonlinear, active, and tunable metasurfaces for advanced electromagnetics applications. *IEEE Access*.

[20] Nemati A., Wang Q., Hong M., Teng J. (2018). Tunable and reconfigurable metasurfaces and metadevices. *Opto-Electronic Advances*.

[21] Staude I., Pertsch T., Kivshar Y. All-dielectric resonant meta-optics goes active. https://arxiv.org/abs/1810.10675.

[22] Abdollahramezani S., Taghinejad H., Fan T., Kiarashinejad Y., Eftekhar A. A., Adibi A. Reconfigurable multifunctional metasurfaces employing hybrid phase-change plasmonic architecture. https://arxiv.org/abs/1809.08907.

[23] Li A., Singh S., Sievenpiper D. (2018). Metasurfaces and their applications. *Nanophotonics*.

[24] Chen H.-T., Padilla W. J., Zide J. M. O., Gossard A. C., Taylor A. J., Averitt R. D. (2006). Active terahertz metamaterial devices. *Nature*.

[25] Ju L., Geng B., Horng J. (2010). Graphene plasmonics for tunable terahertz metamaterials. *Nature Nanotechnology*.

[26] Miao Z., Wu Q., Li X. (2015). Widely tunable terahertz phase modulation with gate-controlled graphene metasurfaces. *Physical Review X*.

[27] Park J., Kang J., Kim S. J., Liu X., Brongersma M. L. (2016). Dynamic reflection phase and polarization control in metasurfaces. *Nano Letters*.

[28] Kafaie Shirmanesh G., Sokhoyan R., Pala R. A., Atwater H. A. (2018). Dual-gated active metasurface at 1550 nm with wide (>300°) phase tunability. *Nano Letters*.

[29] Shrekenhamer D., Chen W.-C., Padilla W. J. (2013). Liquid crystal tunable metamaterial absorber. *Physical Review Letters*.

[30] Tao H., Strikwerda A. C., Fan K., Padilla W. J., Zhang X., Averitt R. D. (2009). Reconfigurable terahertz metamaterials. *Physical Review Letters*.

[31] Zhu W. M., Liu A. Q., Zhang X. M. (2011). Switchable magnetic metamaterials using micromachining processes. *Advanced Materials*.

[32] Cong L., Pitchappa P., Lee C., Singh R. (2017). Active phase transition via loss engineering in a terahertz MEMS metamaterial. *Advanced Materials*.

[33] Ou J.-Y., Plum E., Zhang J., Zheludev N. I. (2013). An electromechanically reconfigurable plasmonic metamaterial operating in the near-infrared. *Nature Nanotechnology*.

[34] Pryce I. M., Aydin K., Kelaita Y. A., Briggs R. M., Atwater H. A. (2010). Highly strained compliant optical metamaterials with large frequency tunability. *Nano Letters*.

[35] Wu P. C., Zhu W., Shen Z. X. (2017). Broadband Wide-Angle Multifunctional Polarization Converter via Liquid-Metal-Based Metasurface. *Advanced Optical Materials*.

[36] Chen H., O'Hara J. F., Azad A. K. (2008). Experimental demonstration of frequency-agile terahertz metamaterials. *Nature Photonics*.

[37] Gu J., Singh R., Liu X. (2012). Active control of electromagnetically induced transparency analogue in terahertz metamaterials. *Nature Communications*.

[38] Shcherbakov M. R., Liu S., Zubyuk V. V. (2017). Ultrafast all-optical tuning of direct-gap semiconductor metasurfaces. *Nature Communications*.

[39] Liu M., Hwang H. Y., Tao H. (2012). Terahertz-field-induced insulator-to-metal transition in vanadium dioxide metamaterial. *Nature*.

[40] Gholipour B., Zhang J., MacDonald K. F., Hewak D. W., Zheludev N. I. (2013). An all-optical, non-volatile, bidirectional, phase-change meta-switch. *Advanced Materials*.

[41] Ren M., Wu W., Cai W., Pi B., Zhang X., Xu J. (2017). Reconfigurable metasurfaces that enable light polarization control by light. *Light: Science & Applications*.

[42] Driscoll T., Kim H., Chae B. (2009). Memory metamaterials. *Science*.

[43] Butakov N. A., Valmianski I., Lewi T. (2017). Switchable plasmonic–dielectric resonators with metal–insulator transitions. *ACS Photonics*.

[44] Chen H., Yang H., Singh R. (2010). Tuning the resonance in high-temperature superconducting terahertz metamaterials. *Physical Review Letters*.

[45] Sautter J., Staude I., Decker M. (2015). Active tuning of all-dielectric metasurfaces. *ACS Nano*.

[46] Duan X., Kamin S., Liu N. (2017). Dynamic plasmonic colour display. *Nature Communications*.

[47] Shadrivov I. V., Kapitanova P. V., Maslovski S. I., Kivshar Y. S. (2012). Metamaterials controlled with light. *Physical Review Letters*.

[48] Xu H., Ma S., Luo W. (2016). Aberration-free and functionality-switchable meta-lenses based on tunable metasurfaces. *Applied Physics Letters*.

[49] Li L., Jun Cui T., Ji W. (2017). Electromagnetic reprogrammable coding-metasurface holograms. *Nature Communications*.

[50] Zhu W., Song Q., Yan L. (2015). A flat lens with tunable phase gradient by using random access reconfigurable metamaterial. *Advanced Materials*.

[51] Watts C. M., Shrekenhamer D., Montoya J. (2014). Terahertz compressive imaging with metamaterial spatial light modulators. *Nature Photonics*.

[52] Huang Y., Lee H. W., Sokhoyan R. (2016). Gate-tunable conducting oxide metasurfaces. *Nano Letters*.

[53] She A., Zhang S., Shian S., Clarke D. R., Capasso F. (2018). Adaptive metalenses with simultaneous electrical control of focal length, astigmatism, and shift. *Science Advances*.

[54] Xu H., Sun S., Tang S. (2016). Dynamical control on helicity of electromagnetic waves by tunable metasurfaces. *Scientific Reports*.

[55] Li A., Kim S., Luo Y., Li Y., Long J., Sievenpiper D. F. (2017). High-power transistor-based tunable and switchable metasurface absorber. *IEEE Transactions on Microwave Theory and Techniques*.

[56] Zhu B., Feng Y., Zhao J., Huang C., Jiang T. (2010). Switchable metamaterial reflector/absorber for different polarized electromagnetic waves. *Applied Physics Letters*.

[57] Tao Z., Wan X., Pan B. C., Cui T. J. (2017). Reconfigurable conversions of reflection, transmission, and polarization states using active metasurface. *Applied Physics Letters*.

[58] Chen H.-T., Padilla W. J., Cich M. J., Azad A. K., Averitt R. D., Taylor A. J. (2009). A metamaterial solid-state terahertz phase modulator. *Nature Photonics*.

[59] Forouzmand A., Salary M. M., Inampudi S., Mosallaei H. (2018). A tunable multigate indium-tin-oxide-assisted all-dielectric metasurface. *Advanced Optical Materials*.

[60] Jun Y. C., Reno J., Ribaudo T. (2013). Epsilon-near-zero strong coupling in metamaterial-semiconductor hybrid structures. *Nano Letters*.

[61] Liberal I., Li Y., Engheta N. (2018). Reconfigurable epsilon-near-zero metasurfaces via photonic doping. *Journal of Nanophotonics*.

[62] Lee J., Jung S., Chen P. (2014). Ultrafast electrically tunable polaritonic metasurfaces. *Advanced Optical Materials*.

[63] Bonaccorso F., Sun Z., Hasan T., Ferrari A. C. (2010). Graphene photonics and optoelectronics. *Nature Photonics*.

[64] Koppens F. H. L., Chang D. E., García de Abajo F. J. (2011). Graphene plasmonics: a platform for strong light–matter interactions. *Nano Letters*.

[65] Novoselov K. S., Geim A. K., Morozov S. V. (2004). Electric field in atomically thin carbon films. *Science*.

[66] Brar V. W., Jang M. S., Sherrott M., Lopez J. J., Atwater H. A. (2013). Highly confined tunable mid-infrared plasmonics in graphene nanoresonators. *Nano Letters*.

[67] Fang Z., Wang Y., Schlather A. E. (2014). Active tunable absorption enhancement with graphene nanodisk arrays. *Nano Letters*.

[68] Thongrattanasiri S., Koppens F. H. L., García de Abajo F. J. (2012). Complete optical absorption in periodically patterned graphene. *Physical Review Letters*.

[69] Lee S. H., Choi M., Kim T.-T. (2012). Switching terahertz waves with gate-controlled active graphene metamaterials. *Nature Materials*.

[70] Shi S., Zeng B., Han H. (2014). Optimizing broadband terahertz modulation with hybrid graphene/metasurface structures. *Nano Letters*.

[71] Mou N., Sun S., Dong H. (2018). Hybridization-induced broadband terahertz wave absorption with graphene metasurfaces. *Optics Express*.

[72] Kim T.-T., Kim H., Kenney M. (2018). Amplitude modulation of anomalously refracted terahertz waves with gated-graphene metasurfaces. *Advanced Optical Materials*.

[73] Kim T.-T., Oh S. S., Kim H.-D. (2017). Electrical access to critical coupling of circularly polarized waves in graphene chiral metamaterials. *Science Advances*.

[74] Zhang Y., Feng Y., Zhao J., Jiang T., Zhu B. (2017). Terahertz beam switching by electrical control of graphene-enabled tunable metasurface. *Scientific Reports*.

[75] Zeng B., Huang Z., Singh A. (2018). Hybrid graphene metasurfaces for high-speed mid-infrared light modulation and single-pixel imaging. *Light: Science & Applications*.

[76] Dabidian N., Dutta-Gupta S., Kholmanov I. (2016). Experimental demonstration of phase modulation and motion sensing using graphene-integrated metasurfaces. *Nano Letters*.

[77] Yao Y., Shankar R., Kats M. A. (2014). Electrically tunable metasurface perfect absorbers for ultrathin mid-infrared optical modulators. *Nano Letters*.

[78] Qu C., Ma S., Hao J. (2015). Tailor the functionalities of metasurfaces based on a complete phase diagram. *Physical Review Letters*.

[79] Sherrott M. C., Hon P. W. C., Fountaine K. T. (2017). Experimental demonstration of >230° phase modulation in gate-tunable graphene-gold reconfigurable mid-infrared metasurfaces. *Nano Letters*.

[80] Degl’Innocenti R., Jessop D. S., Shah Y. D. (2014). Low-bias terahertz amplitude modulator based on split-ring resonators and graphene. *ACS Nano*.

[81] Yu S., Wu X., Wang Y., Guo X., Tong L. (2017). 2D materials for optical modulation: challenges and opportunities. *Advanced Materials*.

[82] Forouzmand A., Salary M. M., Kafaie Shirmanesh G., Sokhoyan R., Atwater H. A., Mosallaei H. (2019). Tunable all-dielectric metasurface for phase modulation of the reflected and transmitted light via permittivity tuning of indium tin oxide. *Journal of Nanophotonics*.

[83] Babicheva V. E., Boltasseva A., Lavrinenko A. V. (2015). Transparent conducting oxides for electro-optical plasmonic modulators. *Journal of Nanophotonics*.

[84] Savo S., Shrekenhamer D., Padilla W. J. (2014). Liquid crystal metamaterial absorber spatial light modulator for THz applications. *Advanced Optical Materials*.

[85] Franklin D., Chen Y., Vazquez-Guardado A. (2015). Polarization-independent actively tunable colour generation on imprinted plasmonic surfaces. *Nature Communications*.

[86] Hsieh C., Pan R., Tang T., Chen H., Pan C. (2006). Voltage-controlled liquid-crystal terahertz phase shifter and quarter-wave plate. *Optics Expresss*.

[87] Karim M. F., Liu A. Q., Alphones A., Yu A. B. (2006). A tunable bandstop filter via the capacitance change of micromachined switches. *Journal of Micromechanics and Microengineering*.

[88] Liu A. Q., Zhu W. M., Tsai D. P., Zheludev N. I. (2012). Micromachined tunable metamaterials: a review. *Journal of Optics*.

[89] Fu Y. H., Liu A. Q., Zhu W. M. (2011). A micromachined reconfigurable metamaterial via reconfiguration of asymmetric split-ring resonators. *Advanced Functional Materials*.

[90] Ma F., Lin Y., Zhang X., Lee C. (2014). Tunable multiband terahertz metamaterials using a reconfigurable electric split-ring resonator array. *Light: Science & Applications*.

[91] Zheludev N. I., Plum E. (2016). Reconfigurable nanomechanical photonic metamaterials. *Nature Nanotechnology*.

[92] Reeves J. B., Jayne R. K., Stark T. J., Barrett L. K., White A. E., Bishop D. J. (2018). Tunable infrared metasurface on a soft polymer scaffold. *Nano Letters*.

[93] Zhu L., Kapraun J., Ferrara J., Chang-Hasnain C. J. (2015). Flexible photonic metastructures for tunable coloration. *Optica*.

[94] Tseng M. L., Yang J., Semmlinger M., Zhang C., Nordlander P., Halas N. J. (2017). Two-dimensional active tuning of an aluminum plasmonic array for full-spectrum response. *Nano Letters*.

[95] Kamali S. M., Arbabi E., Arbabi A., Horie Y., Faraon A. (2016). Highly tunable elastic dielectric metasurface lenses. *Laser & Photonics Reviews*.

[96] Ee H., Agarwal R. (2016). Tunable Metasurface and Flat Optical Zoom Lens on a Stretchable Substrate. *Nano Letters*.

[97] Kasirga T. S., Ertas Y. N., Bayindir M. (2009). Microfluidics for reconfigurable electromagnetic metamaterials. *Applied Physics Letters*.

[98] Sun S., Yang W., Zhang C. (2018). Real-time tunable colors from microfluidic reconfigurable all-dielectric metasurfaces. *ACS Nano*.

[99] Padilla W. J., Taylor A. J., Highstrete C., Lee M., Averitt R. D. (2006). Dynamical electric and magnetic metamaterial response at terahertz frequencies. *Physical Review Letters*.

[100] Cong L., Srivastava Y. K., Zhang H., Zhang X., Han J., Singh R. (2018). All-optical active THz metasurfaces for ultrafast polarization switching and dynamic beam splitting. *Light: Science & Applications*.

[101] Zhao Q., Zhou J., Zhang F. L., Lippens D. (2009). Mie resonance-based dielectric metamaterials. *Materials Today*.

[102] Rensberg J., Zhang S., Zhou Y. (2016). Active optical metasurfaces based on defect-engineered phase-transition materials. *Nano Letters*.

[103] Wang Q., Rogers E. T., Gholipour B. (2016). Optically reconfigurable metasurfaces and photonic devices based on phase change materials. *Nature Photonics*.

[104] Liu L., Kang L., Mayer T. S., Werner D. H. (2016). Hybrid metamaterials for electrically triggered multifunctional control. *Nature Communications*.

[105] Pitchappa P., Kumar A., Prakash S., Jani H., Venkatesan T., Singh R. (2019). Chalcogenide phase change material for active terahertz photonics. *Advanced Materials*.

[106] Yin X., Steinle T., Huang L. (2017). Beam switching and bifocal zoom lensing using active plasmonic metasurfaces. *Light: Science & Applications*.

[107] Hosseini P., Wright C. D., Bhaskaran H. (2014). An optoelectronic framework enabled by low-dimensional phase-change films. *Nature*.

[108] Kats M. A., Blanchard R., Genevet P. (2013). Thermal tuning of mid-infrared plasmonic antenna arrays using a phase change material. *Optics Expresss*.

[109] Ding F., Zhong S., Bozhevolnyi S. I. (2018). Vanadium dioxide integrated metasurfaces with switchable functionalities at terahertz frequencies. *Advanced Optical Materials*.

[110] Jin B., Zhang C., Engelbrecht S. (2010). Low loss and magnetic field-tunable superconducting terahertz metamaterial. *Optics Express*.

[111] Komar A., Paniagua-Domínguez R., Miroshnichenko A. (2018). Dynamic beam switching by liquid crystal tunable dielectric metasurfaces. *ACS Photonics*.

[112] Sievenpiper D. F., Schaffner J. H., Song H. J., Loo R. Y., Tangonan G. (2003). Two-dimensional beam steering using an electrically tunable impedance surface. *IEEE Transactions on Antennas and Propagation*.

[113] Xu H., Tang S., Ma S. (2016). Tunable microwave metasurfaces for high-performance operations: dispersion compensation and dynamical switch. *Scientific Reports*.

[114] Chen K., Feng Y., Monticone F. (2017). A reconfigurable active huygens' metalens. *Advanced Materials*.

[115] Cui T. J., Qi M. Q., Wan X., Zhao J., Cheng Q. (2014). Coding metamaterials, digital metamaterials and programmable metamaterials. *Light: Science & Applications*.

[116] Cui T. J. (2017). Microwave metamaterials—from passive to digital and programmable controls of electromagnetic waves. *Journal of Optics*.

[117] Huang C., Yang J., Wu X. (2018). Reconfigurable metasurface cloak for dynamical electromagnetic illusions. *ACS Photonics*.

[118] Zhang X. G., Tang W. X., Jiang W. X. (2018). Light-controllable digital coding metasurfaces. *Advanced Science*.

[119] Yan L. B., Zhu W. M., Wu P. C. (2017). Adaptable metasurface for dynamic anomalous reflection. *Applied Physics Letters*.

[120] Yan L., Zhu W., Karim M. F. (2018). Arbitrary and independent polarization control in situ via a single metasurface. *Advanced Optical Materials*.

[121] Ding L., Luo X., Cheng L. (2018). Electrically and thermally tunable smooth silicon metasurfaces for broadband terahertz antireflection. *Advanced Optical Materials*.

[122] Yang H., Yu T., Wang Q., Lei M. (2017). Wave manipulation with magnetically tunable metasurfaces. *Scientific Reports*.

[123] Liu Y. J., Si G. Y., Leong E. S. P., Xiang N., Danner A. J., Teng J. H. (2012). Light-driven plasmonic color filters by overlaying photoresponsive liquid crystals on gold annular aperture arrays. *Advanced Materials*.

[124] Afridi A., Canet-Ferrer J., Philippet L., Osmond J., Berto P., Quidant R. (2018). Electrically driven varifocal silicon metalens. *ACS Photonics*.

[125] Zhang L., Chen X. Q., Liu S. (2018). Space-time-coding digital metasurfaces. *Nature Communications*.

[126] Dai J. Y., Zhao J., Cheng Q. (2018). Independent control of harmonic amplitudes and phases via a time-domain digital coding metasurface. *Light: Science & Application*.

[127] Shaltout A., Kildishev A., Shalaev V. (2015). Time-varying metasurfaces and Lorentz non-reciprocity. *Optical Materials Express*.

[128] Assouar B., Liang B., Wu Y., Li Y., Cheng J., Jing Y. (2018). Acoustic metasurfaces. *Nature Reviews Materials*.

[129] Li Y., Shen C., Xie Y. (2017). Tunable asymmetric transmission via lossy acoustic metasurfaces. *Physical Review Letters*.

[130] Chen S., Fan Y., Fu Q. (2018). A review of tunable acoustic metamaterials. *Applied Sciences*.

[131] Cummer S. A., Christensen J., Alù A. (2016). Controlling sound with acoustic metamaterials. *Nature Reviews Materials*.

[132] Xie B., Tang K., Cheng H., Liu Z., Chen S., Tian J. (2017). Coding acoustic metasurfaces. *Advanced Materials*.

[133] Li Y., Shen X., Wu Z. (2015). Temperature-dependent transformation thermotics: from switchable thermal cloaks to macroscopic thermal diodes. *Physical Review Letters*.

[134] Nguyen D. M., Xu H., Zhang Y., Zhang B. (2015). Active thermal cloak. *Applied Physics Letters*.

